# Pioneering Endoscopic Submucosal Dissection in the West: Technique Introduction With a Comprehensive Case Report on the First Application for Oropharyngeal Squamous Cell Carcinoma

**DOI:** 10.14309/crj.0000000000001956

**Published:** 2026-01-14

**Authors:** Ken Namikawa, Einar Stefan Bjornsson, Helgi Kristinn Sigmundsson, Geir Tryggvason, Sigfus Thor Nikulasson, Jon Gunnlaugur Jonasson, Magnus Konradsson

**Affiliations:** 1 Department of Gastroenterology, Landspítali–The National University Hospital of Iceland, Reykjavík, Iceland; 2 Department of Gastroenterology, Cancer Institute Hospital, Japanese Foundation for Cancer Research, Tokyo, Japan; 3 Faculty of Medicine, University of Iceland, Reykjavik, Iceland; 4 Department of Otorhinolaryngology, Landspítali–The National University Hospital of Iceland, Reykjavik, Iceland; 5 Department of Pathology, Landspítali–The National University Hospital of Iceland, Reykjavík, Iceland

**Keywords:** pharyngeal endoscopic submucosal dissection, pharyngeal cancer, oropharyngeal squamous cell carcinoma, the West, early detection

## Abstract

Endoscopic submucosal dissection (ESD) for pharyngeal squamous cell carcinoma (SCC) has predominantly been performed in Asia but interest in this procedure is growing in the West. This report details the first known Western application of ESD for oropharyngeal SCC. A Caucasian female with superficial oropharyngeal SCC detected during endoscopic surveillance post-ESD for esophageal SCC underwent successful en-bloc resection without complications. Pathology confirmed SCC in situ with tumor-free margins. This case underscores the importance of mindset to include the pharynx in upper gastrointestinal endoscopy during surveillance within the gastroenterologist's domain and the potential for broader application of ESD in the West.

## INTRODUCTION

Pharyngeal squamous cell carcinoma (SCC) patients often present with advanced stage malignancy, which is associated with poor prognosis.^1,2^ By contrast, superficial pharyngeal SCC is potentially curable with endoscopic submucosal dissection (ESD), which offers a minimally invasive and function-preserving local resection.^3–5^ ESD for pharyngeal SCC is established in Asia, but despite a growing interest, to the best of our knowledge, the application of ESD for oropharyngeal SCC in the West has not yet been reported.^3–7^ This paper details the use of ESD technique as treatment with intention for cure of superficial pharyngeal cancer and its outcome through the first case report for oropharyngeal SCC in the West.

## CASE REPORT

**Figure 1.**
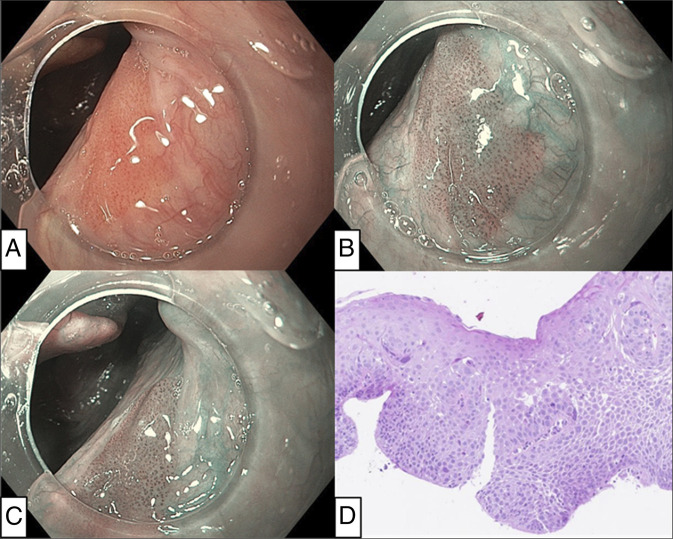
Endoscopic and histopathological images of oropharyngeal squamous cell carcinoma at diagnosis. (A) The gastroscope with attachment cap revealed a reddish flat lesion in white light imaging. (B) The lesion was more clearly visualized under narrow band imaging. (C) The location of the tumor was at the edge of the right palatopharyngeal arch, according to a relative positional relation to the epiglottis. (D) The biopsy specimen showed severe atypia amounting to conventional squamous cell carcinoma in situ (hematoxylin & eosin stain).

A 74-year-old White female, with a history of alcohol overconsumption (moderate to severe alcohol disorder with episodes of binging over 40-year periods) and 40 pack-year smoking, had been under surveillance for 5 years after ESD for esophageal SCC. A tumor was identified on the right side of the oropharynx by surveillance upper gastrointestinal (GI) endoscopy, and the biopsy showed conventional SCC (Figure 1).

**Figure 2.**
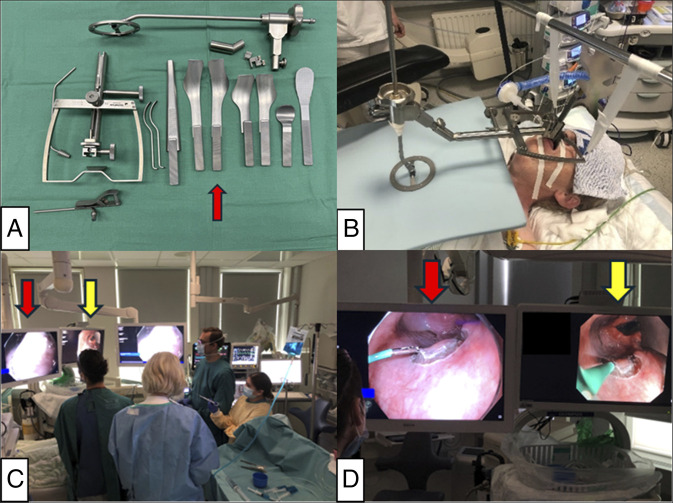
Preoperative and intraoperative photographs of procedure-setup. (A) FK-WO Laryngo-Pharyngoscope Retractor system. The blade for right-sided tongue base lesions (red arrow) was chosen in this case. (B) Patient positioning and laryngo-pharyngoscope retractor setup. A transoral retractor provided wide exposure of the pharynx. (C) Endoscopy room setup during the procedure. The primary display monitor (red arrow) for transoral gastroscopy and the secondary display monitor (yellow arrow) for transnasal ultrathin gastroscopy were placed. (D) The primary display monitor (red arrow) showed a video image from the transoral gastroscope for resection and the secondary display monitor (yellow arrow) showed the image from the transnasal ultrathin gastroscope for traction. FK-WO, Feyh-Kastenbauer-Weinstein-O’Malley.

**Figure 3.**
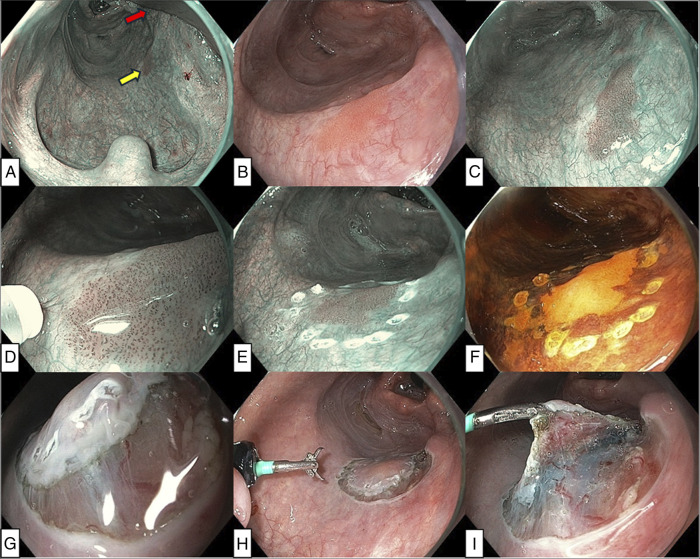
Intraoperative endoscopic images. (A) A wide exposure of the oropharynx was achieved by lifting the larynx by the retractor blade (red arrow), providing adequate working space and optimal visualization of the lesion (yellow arrow). (B) The entire form of the tumor was observed as a 12-mm sized reddish flat lesion. (C) The lesion was visualized more clearly as a brownish area under narrow band imaging (NBI). (D) Marking was performed under near-focus NBI. Irregular intraepithelial papillary capillary loops were observed in the lesion, corresponding to type B1 vessels according to the magnifying endoscopic classification of the Japan Esophageal Society for esophageal squamous cell carcinoma.^13^ (E) Marking dots were placed around the lesion. (F) The border of the lesion was confirmed by iodine staining. The lesion was visualized as an unstained area. (G) Mucosal incision outside the marking dots. The typically thin mucosal epithelium of the pharynx was observed. (H) After circumferential incision, transnasal endoscopy with an ultra-slim gastroscope was performed to grasp the specimen for traction. (I) Double-scope traction method provided a better visualization of the layer for dissection.^6^

**Figure 4.**
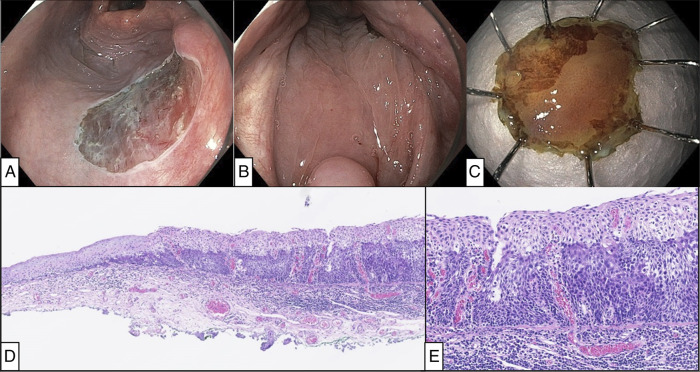
Postoperative endoscopic and histopathological images. (A) En block resection was achieved. (B) The view of the pharynx without the retractor. Most of the mucosal defect was covered by the lateral wall of the oropharynx. (C) Tumor was visualized as an unstained area by iodine staining in ESD specimen. (D) Histology of the resection specimen showing conventional squamous cell carcinoma in situ to the right and normal epithelium towards the left. Chronic inflammatory infiltration was present in underlying stroma (hematoxylin & eosin stain). (E) Higher power picture of conventional squamous cell carcinoma in situ in the resection sample showing full-thickness epithelial atypia. Lymphocytic infiltration was present in the underlying stroma, but no evidence of invasive tumor (hematoxylin & eosin stain). ESD, endoscopic submucosal dissection.

ESD was performed with assistance of a surgical retractor under general anesthesia. After creation of marking-dots around the lesion, a lifting solution was injected into the subepithelium. Subsequently, circumferential mucosal incision was performed with the ESD knife, and subepithelial dissection was performed with the advantage of a double-scope traction method using a regular transoral scope for resection and an ultrathin transnasal scope for traction (Figures 2 and 3).^4^ During and after completion of ESD, hemostasis was provided by a DualKnife with a retracted tip, and major bleeding did not occur. Details of the ESD procedure are presented in Figures 2 and 3 and Table 1. Postoperative pain was well controlled with oral route acetaminophen 4 × 1,000 mg/d and extended-release morphine 10 mg/d. Oral intake could be initiated on postoperative day (POD) 0. She was discharged on POD 1, and no delayed complications occurred. After discharge, pain management was continued with the same oral medications, which she discontinued on POD 7. The final pathology diagnosis from ESD specimen was pharyngeal SCC (conventional type) in situ with tumor-free margin (0.3-mm vertical and 2-mm lateral margin) (Figure 4).

**Table 1.** Devices, equipment, and electrosurgical setting for endoscopic submucosal dissection

Characteristics	
Scope	• Dual Focus gastroscope (EVIS EXERA III GIF-HQ190; Olympus Co. Ltd., Tokyo, Japan) • Ultra-slim gastroscope (EVIS EXERA III GIF-XP190N; Olympus)
Attachment of scope	Mounted distal attachment cap (D-201-11802; Olympus)
Electrical surgical unit	VIO 3 (ERBE Elektromedizin, Tübingen, Germany)
Marking	Forced Coagulation mode (Effect 2)
Mucosal incision	Endo Cut I mode (Effect 1, Duration 2, Interval 5)
Dissection	• Endo Cut I mode (Effect 1, Duration 2, Interval 5) • Precise SECT mode (Effect 3)
Hemostasis	Soft Coagulation mode (Effect 5)
ESD knife	Dualknife J (KD-650U; Olympus)
Injection solutions	• 0.9% sodium chloride • 0.001% epinephrine • 0.01% methylene blue dye
Retractor	FK-WO Laryngo-Pharyngoscope Retractor (Olympus Europa SE & Co. KG, Hamburg, Germany)

ESD, endoscopic submucosal dissection; FK-WO, Feyh-Kastenbauer-Weinstein-O’Malley.

## DISCUSSION

A systematic review and meta-analysis by Kamal et al found all the included studies on ESD for pharyngeal SCC were from Asia, highlighting the need for studies from Western countries to validate their findings.^5^ To date, only 2 endoscopically treated pharyngeal cancers have been reported in the West: 1 ESD in France and 1 endoscopic mucosal resection in the United States for hypopharyngeal SCCs.^7,8^

This disparity may stem from lack of screening programs, routine examinations in the pharynx, and experience in diagnosing and treating superficial pharyngeal SCC. Gastroenterologists typically exclude the examination in the pharynx from upper GI endoscopy, assuming it beyond their purview.^9^ However, we believe that early detection of pharyngeal cancers should fall within the gastroenterologist's domain, given its association with esophageal SCC, as both share etiological factors such as alcohol consumption and smoking.^1,9,10^ Gastroenterologists diagnose and treat esophageal SCCs using a high-resolution endoscope, which could be effectively extended to the pharynx.

For successful pharyngeal cancer screening, narrow band imaging (NBI) is efficient since the established NBI-findings in esophageal SCC are applicable to the pharynx.^10,11^ Adequate sedation is essential for proper examination to manage swallowing and avoid eliciting the gag reflex. It is also important to be aware of potential pitfalls due to the blind spots in the pharynx such as epiglottic vallecula, lateral wall of oropharynx, and deep hypopharynx.

ESD offers a high en-block resection rate, which is advantageous over endoscopic mucosal resection.^5^ Although comparative studies with minimally invasive surgical treatment modalities are lacking, potential advantages of ESD include cost effectiveness, and precise function sparing resection due to the maneuverability of the flexible endoscope.^5^

Nevertheless, there are some special properties for ESD in the pharynx compared with other GI tract areas that are important to note. It carries a risk of respiratory failure due to laryngeal edema or delayed bleeding. In addition, it demands unique technical expertise in the limited working-space and requires general anesthesia and larynx-lifting.

The current case demonstrated the feasibility of early-stage SCC detection by a meticulous endoscopic inspection, integrating risk factor awareness; prior esophageal SCC, and excessive alcohol/tobacco consumption. Although routine screening endoscopic investigations in the pharynx may be impractical in the West, targeted evaluation in high-risk individuals could be valuable. Oropharyngeal lesions are frequently overlooked in upper GI endoscopy due to anatomical complexity, as shown in Figure 4, but the use of NBI, an attachment cap, and awareness of blind spots helped early detection in our case, demonstrating typical findings of superficial SCC (Figures 1 and 2)^9–11^.

Modifying routines and mindsets to incorporate the pharynx in upper GI endoscopy, based on individual risk profiles, may be more crucial and challenging than the actual process of learning how to recognize pharyngeal SCC.

ESD access in the pharynx requires larynx-lifting, often achieved with support from otolaryngologists/head and neck surgeons, and specialized retractor.^3–5^ In this case, A common retractor system for head and neck surgery was successfully applied by endoscopists and anesthesiologist, allowing for an efficient procedure (Figure 1). Double-scope traction method efficiently facilitated dissection (Figure 3)^4^. ESD allowed for resection without excess margin, minimizing mucosal defect and muscularis damage, as shown in Figure 4. This probably contributed to rapid postoperative recovery.

In conclusion, early detection of pharyngeal SCC should preferably be incorporated in the objectives of upper GI endoscopy when indicated. ESD for pharyngeal SCC may offer potential benefits in patient management; however, further studies from the West are needed to validate current findings. As Western centers gain more experience with ESD in other GI locations, the time has come to consider broader implementation into the pharynx.^12,13^

## DISCLOSURES

Author contributions: K. Namikawa and M. Konradsson substantially contributed to the conception of the work. K. Namikawa, HK Sigmundsson, G. Tryggvason, and M. Konradsson made contribution to collecting and analysis in clinical data. ST Nikulasso and JG Jonasson made contribution to collecting and analysis in pathological data. K. Namikawa made the manuscript drafting ES Bjornsson and M. Konradsson made substantial contribution to the manuscript editing. All authors critically reviewed and revised the manuscript draft and approved the final version for submission. K. Namikawa is the article guarantor.

Financial disclosure: None to report.

Previous presentation: This case was presented at the European Society for Diseases of the Esophagus (ESDE) congress 2025; May 11–13, 2025; Amsterdam, the Netherlands.

Informed consent was obtained for this case report.

## ABBREVIATIONS:

EMR: endoscopic mucosal resection
ESD: endoscopic submucosal dissection
FK-WO: Feyh-Kastenbauer-Weinstein-O’Malley
GI: gastrointestinal
NBI: narrow band imaging
POD: postoperative day
SCC: squamous cell carcinoma
